# Correlative imaging and histopathology of a complicated sinonasal teratocarcinosarcoma

**DOI:** 10.4102/sajr.v27i1.2548

**Published:** 2023-01-30

**Authors:** Tineke van Zyl, Leon Janse van Rensburg, Komeela Naidoo, Marc Merven, Johan F. Opperman

**Affiliations:** 1 Department of Maxillofacial Radiology, Faculty of Dentistry, University of the Western Cape, Cape Town, South Africa; 2 Department of Medical Imaging and Clinical Oncology, Faculty of Medicine and Health Sciences, Stellenbosch University, Cape Town, South Africa; 3 Department of Radiation Oncology, Faculty of Medical Imaging and Clinical Oncology, Stellenbosch University, Cape Town, South Africa; 4 Department of Ear, Nose and Throat, Faculty of Medicine, Stellenbosch University, Cape Town, South Africa; 5 Department of Oral and Maxillofacial Pathology, Faculty of Dentistry, University of the Western Cape, Cape Town, South Africa; 6 Department of Oral and Maxillofacial Pathology, National Health Laboratory Service, Cape Town, South Africa

**Keywords:** Sinonasal teratocarcinosarcoma, histopathology, computerised tomography, magnetic resonance imaging, intracranial, diffusion-weighted imaging

## Abstract

Sinonasal teratocarcinosarcoma (SNTCS) is a highly malignant and rare tumour characterised by a complex admixture of teratomatous and carcinosarcomatous components. In the head and neck area, it almost exclusively occurs in the sinonasal cavities; however, rare instances of nasopharyngeal and oral cavity involvement have been reported, with fewer than 100 cases reported in the literature.

**Contribution:**

The contributed case involves the correlative CT, MRI and histopathology of a sinonasal teratocarcinosarcoma with intracranial involvement.

## Introduction

Sinonasal teratocarcinosarcoma (SNTCS) is a rare tumour characterised by the presence of benign and malignant epithelial, mesenchymal and neural components.^1^ In view of its complex histological architecture, SNTCS has also previously been classified as malignant *teratoma, blastoma* or *mixed mesodermal tumour*.^2^ It was first described by Shanmugaratnam et al. in 1983, but only appropriately termed teratocarcinosarcoma by Heffner and Hyams in 1984.^2^ Male patients are affected more than female patients, with the head and neck area, nasal cavity and paranasal sinuses most commonly involved.^3,4^ Frequent symptoms include nasal obstruction with epistaxis, atypical facial pain, exophthalmos, headaches, epiphora and anosmia.^4,5^ A mean survival rate of 1.7 years is reported with a 60% mortality rate within three years.^6^

Intracranial extension and intracranial involvement are rarely observed. A series of 86 cases of SNTCS reviewed by Misra et al. reported 20.9% of cases demonstrating intracranial, cribriform plate and anterior cranial fossa involvement.^7^ Because of its anatomical location, an accurate pre-operative diagnosis is essential, especially to demonstrate intracranial extension, which may be occult on CT; MRI is, however, superior to CT for detecting intracranial extension.

Owing to its heterogeneous histological appearance, adequate sampling and recognition of all the components of this type of tumour are needed for its correct diagnosis. Inadequate sampling may lead to erroneous diagnoses of olfactory neuroblastoma, squamous cell carcinoma, undifferentiated carcinoma, adenocarcinoma, malignant salivary gland–type tumours and adenosquamous carcinoma.^5,8^

This article describes the CT and MRI findings as well as the histopathology of a rare case of a SNTCS, with the epicentre in the left nasal cavity involving the left maxillary sinus, with intracranial extension. To the authors’ knowledge, this is the first case described in South Africa.

## Case presentation

A 44-year-old man presented with a 3-month history of a rapidly growing left-sided nasal mass. He complained of recurrent epistaxis, blocked nose and generalised headaches, concentrated mostly on the left side of his face; however, there were no visual or olfactory disturbances. His medical history included hypertension and previous pulmonary tuberculosis. Nasal endoscopic examination revealed a distended left nostril with a protruding, friable, haemorrhagic, exophytic, foul-smelling mass. The rest of the clinical examination was unremarkable.

Brush cytology of the lesion showed atypical malignant epithelial cells morphologically suggestive of a carcinomatous lesion. Based on the cytological features and radiological imaging, a provisional diagnosis of a Schneiderian papilloma with malignant transformation, sinonasal neuroendocrine carcinoma (SNEC), sinonasal undifferentiated carcinoma (SNUC), sinonasal adenocarcinoma or a malignant salivary gland neoplasm was considered.

Following demonstration of an intracranial component on MRI, a decision was made to treat surgically with a combined rhinology and neurosurgery approach. The intracranial tumour was removed first, followed by a transnasal endoscopic procedure. Histology revealed a SNTCS. Postoperatively, the patient developed a brain abscess with associated seizures and convulsions. The brain abscess was drained, but the patient subsequently demised as a result of rapid disease progression.

### Radiological imaging

**FIGURE 1:**
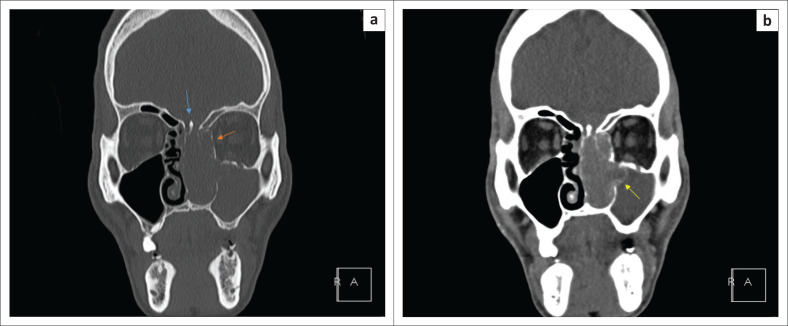
(a) Coronal CT bone window shows an expansile, polypoid mass in the left nasal cavity. Superiorly, the cribriform plate, olfactory recess and fovea ethmoidalis are eroded and widened (blue arrow). The nasal septum is deviated to the right. The left anterior ethmoid and frontal sinus air cells are opacified. There is convex bulging of the lamina papyracea, which is eroded (orange arrow). The infundibulum and middle meatus are eroded and widened. The left maxillary is opacified. The hard palate is eroded. (b) Coronal CT soft tissue contrast-enhanced image shows a uniformly contrast-enhancing polypoid tumour in the left nasal cavity, extending to the cribriform plate, olfactory recess and ethmoid sinus. No demonstrable intracranial extension. The left maxillary sinus outflow tract is widened and obstructed by a tumour extending into the infundibulum and middle meatus with a resultant effusion (yellow arrow).

A CT scan revealed a soft tissue mass with its epicentre in the left nasal cavity, measuring approximately 89 mm × 30 mm × 46 mm in diameter (anteroposterior × transverse × craniocaudal). Anteriorly, the mass projected through the nostril and posteriorly extended to the posterior choana. Superiorly, the mass extended to the cribriform plate with bony demineralisation. Bulging and demineralisation of the medial wall of the left orbit was present, but no intra-orbital involvement was evident (Figure 1).

**FIGURE 2:**
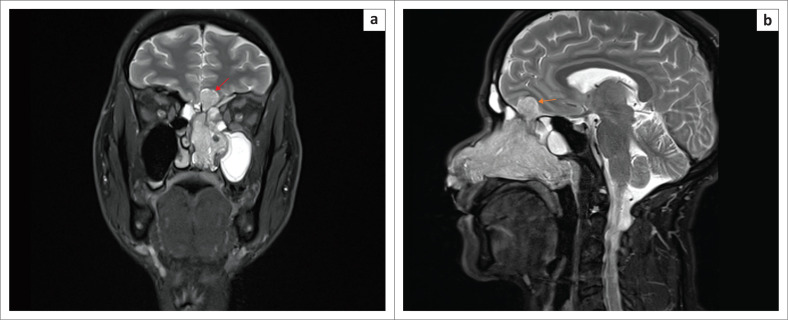
(a) Coronal T2 turbo spin echo (TSE) with fat saturation (FS) MRI. There is an expansile T2 solid hyperintense polypoid mass extending intracranially into the anterior cranial fossa with mild mass effect on the inferior left frontal lobe (red arrow). The maxillary sinus is obstructed with a resultant effusion. (b) Sagittal T2 turbo spin echo (TSE) with fat saturation (FS) MRI image shows the antero-posterior extent of the mass, the extradural intracranial component (orange arrow) and obstructed frontal and sphenoid sinuses.

**FIGURE 3:**
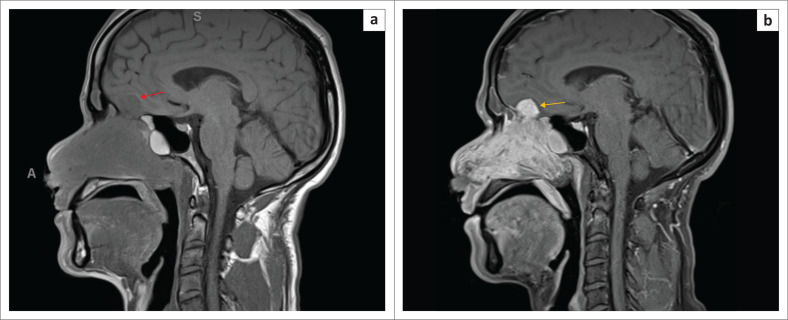
(a) T1 turbo spin echo (TSE) sagittal MR image shows a homogeneously mass, iso-intense to grey matter and muscle (red arrow). (b) Sagittal T1 turbo spin echo (TSE) with fat saturation (FS) with gadolinium-enhanced MR image shows a diffusely heterogeneous enhancing tumour in the left nasal cavity that transgresses the cribriform plate extending intracranially (orange arrow).

The MRI showed a T2 hyperintense, T1 isointense and post–contrast enhancing mass in the left nasal cavity. The nasal septum was deviated to the right, with obstruction of the maxillary sinus. Superiorly, the mass extended into the left anterior cranial fossa and frontal lobe with mild mass effect but no peritumoural oedema (Figures 2 and 3). The lateral ventricles, basal cisterns, cerebellum and brainstem were normal.

**FIGURE 4:**
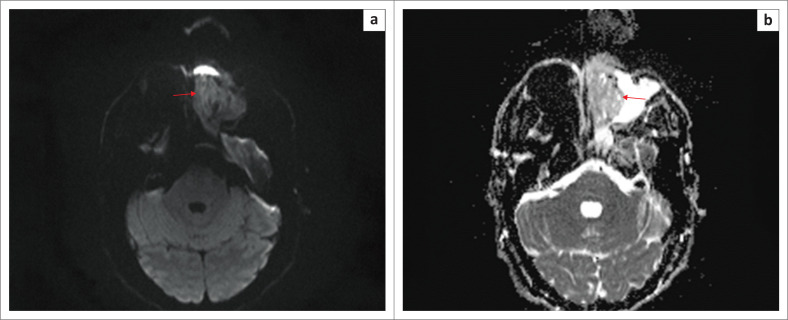
(a) Diffusion-weighted imaging B-1000/mm. The mass shows restricted diffusion (red arrow). There is an area of susceptibility artefact anteriorly and at the posterior skull base. (b) Axial ADC map MRI image shows a lobulated left nasal mass with restricted diffusion and small foci of T2 shine-through, ascribed to small foci of intratumoural cysts or necrosis (red arrow).

At DWI B-1000/mm, the mass showed increased signal. There were areas of susceptibility artefact anteriorly and at the posterior skull base (Figure 4). The axial ADC map showed a lobulated left nasal mass with restricted diffusion and small foci of T2 shine-through, ascribed to small foci of intratumoural cysts or necrosis (Figure 4).

### Histopathology

**FIGURE 5:**
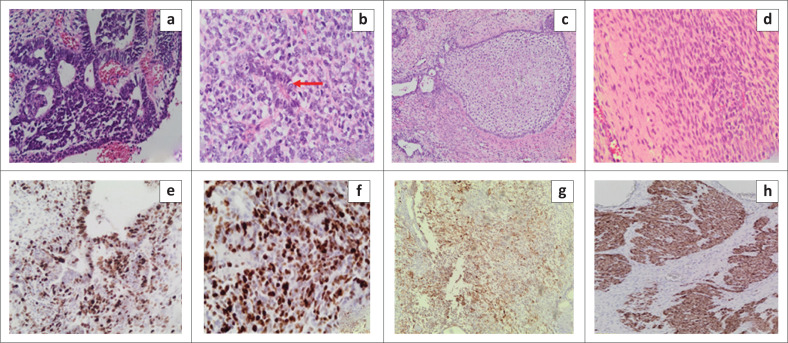
Pathological examination showed (a) adenocarcinomatous glands; (b) adjacent primitive neuroepithelium with rosette-like structures; and (c) islands of ‘foetal’ clear-cell squamous cells. (d) Shows a malignant spindled smooth muscle component with numerous atypical mitotic figures. (e) Proliferation index (ki67) > 70%. (f and g) Immunoreactivity of neuroepithelium with S100 and CD56, respectively, and (h) desmin positivity demonstrating smooth muscle differentiation.

The surgically resected specimen showed a necrotic, friable haemorrhagic tissue mass. Microscopic examination revealed fragments of inflamed sinonasal mucosa with foci of atypical glandular structures, composed of pleomorphic cuboidal to columnar epithelium, demonstrating irregular hyperchromatic nuclei and abundant cytoplasm (Figure 5a). Adjacent to the malignant glands, islands of primitive-appearing round-to-oval immature-appearing cells were noted, morphologically reminiscent of olfactory neuroblastoma, representing the neuroepithelial component (Figure 5b). Mitotic figures and rosette-like structures were present, admixed with numerous islands comprising ‘foetal’ clear squamous cells (Figure 5c). Osseous and cartilaginous differentiation was absent, but sarcomatous areas were evident, composed of fascicles of pleomorphic spindle cells (Figure 5d).

Immunohistochemical staining revealed a high proliferation index in the atypical glands and primitive round cells. The adenocarcinoma component showed immunoreactivity with cytokeratin AE1 and AE3 (not shown). The primitive neuroepithelium was positive with S100, CD56 and neuron-specific enolase (NSE) (Figure 5f and g), as well as with neuroendocrine markers, synaptophysin and chromogranin (not shown), while the clear-cell squamous cells were immunoreactive to CD99 (not shown). The spindle cell sarcomatous component was positive for smooth muscle markers, desmin and α-smooth muscle actin, representing a leiomyosarcoma (Figure 5d and 5h). The immunohistochemical staining pattern and histomorphological features were consistent with the diagnosis of a SNTCS.

## Discussion

Sinonasal teratocarcinosarcoma is a rare and aggressive tumour showing combined histological features of a carcinosarcoma and a teratoma.^2^ The histogenesis of SNTCS is debatable. The presence of neural tissue in these neoplasms raises the possibility that the origin may be in some way related to the olfactory membrane, or alternatively to the sinonasal membrane, as it also develops from the olfactory membranes. Other theories propose germ cell origin or pluripotential progenitor cell with multidirectional differentiation.^9,10^ In contradiction to malignant gonadal teratomas, which are frequently found in patients at a younger age, SNTCS does not contain histological evidence of embryonal carcinoma, choriocarcinoma or germinomas (seminomas), making germ cell origin for SNTCS unlikely.^9,10,11^

These tumours are more common in adults in the fourth to fifth decade and occur mostly in male patients, with only five cases reported in children.^10^ In a series reported by Smith et al., a total of 9 out of 10 tumours occurred in the nasal cavity with extension to the maxillary sinus, the ethmoid sinus and sphenoid sinus.^12^ A review of the literature revealed 15 cases of SNTCS with intracranial extension between 1998 and 2020.^13,14,15^ The first case of a primary thyroid teratocarcinosarcoma in a 17-year-old male patient was reported by Abayie et al., implying that teratocarcinosarcomas can occur in primary tissues other than the sinonasal tract.^16^

Sinonasal tumours usually lack specific CT and MRI features, and the imaging is similar to common malignant sinus tumours such as olfactory neuroblastomas, small cell carcinomas, immature teratomas, carcinosarcomas and adenocarcinomas. CT findings of a mass widening the olfactory recess and extending to the cribriform plate should alert to the possibility of intracranial extension, and MRI is indicated.^17^ Nguyen reported that F-18 fluorodeoxyglucose (FDG) uptake by normal brain parenchyma may be a pitfall of not detecting intracranial invasion on F18-FDG-positron emission tomography (PET)/CT. An advantage of FDG-PET/CT, however, is that regional and distant metastases can be detected.^17^

The infrequency, complex phenotypic composition and radiological similarities with other malignant sinonasal tumours often lead to misdiagnoses and management difficulties. These tumours may therefore be mistaken for olfactory neuroblastoma because of their neuroectodermal histological component and transgression of the cribriform plate on radiology. Histological features that may aid in the recognition of these tumours are the presence of immature neural tissue and foetal clear-cell epithelial structures admixed with various malignant and mesenchymal elements. A panel of immunohistochemical antibodies – monoclonal and polyclonal – can be utilised to assist in the diagnosis of SNTCS. The glandular and/or epithelial structures show positive immuno-expression with epithelial membrane antigen (EMA) and keratins (CK); the mesenchymal components express various antibodies, for example, vimentin, desmin and alpha smooth muscle actin (α-SMA), the latter two indicating smooth muscle differentiation. Neurofilament (NF), glial fibrillary acid protein (GFAP), neuron-specific enolase (NSE) and S100 protein show the presence of primitive neural elements. CD99 expression is noted in the foetal clear-cell epithelial component while Beta human chorionic gonadotropin (β-HCG) and alpha foetoprotein (AFP) are typically negative, highlighting the absence of germ cell elements.

In summary, SNTCS is an aggressive tumour, exhibiting a highly invasive behaviour and a propensity for recurrence and dissemination. Both CT and MRI can detect the tumour and determine the local extent; MRI is, however, more sensitive for detection of intracranial extension. Total excision of the tumour and aggressive sampling for histopathological examination is necessary to avoid an erroneous diagnosis. Treatment usually involves multimodal surgical resection, with or without adjuvant treatments comprising radiation and chemotherapy.^14,18,19^ Aggressive tumour spread, intracranial extension and high recurrence rate may negatively impact the survival rate.^14,15^ Despite aggressive surgical resection and adjuvant radiation therapy, the prognosis is still poor, as half of the patients with SNTCS succumb to their disease within three years of diagnosis.^15^
